# A case of two ovarian tissue transplantations that led to a biochemical pregnancy in Japan

**DOI:** 10.1111/jog.15192

**Published:** 2022-02-17

**Authors:** Yusuke Sako, Fumi Akitani, Kyoko Shiota, Mikio Momoeda

**Affiliations:** ^1^ Department of Obstetrics and Gynecology St. Luke's International Hospital Tokyo Japan

**Keywords:** case reports, cryopreservation, fertility preservation, laparoscopic surgery, tissue transplantation

## Abstract

Ovarian tissue cryopreservation (OTC) and ovarian tissue transplantation (OTT) are fertility preservation options for prepubertal girls or those in whom cancer treatment cannot be delayed. They are important to increasing number of cancer survivors, but there are very few reports on this topic in Japan. This is the first report of a biochemical pregnancy after OTT in Japan. An 18‐year‐old woman, diagnosed with Ewing sarcoma of the seventh thoracic vertebra, underwent tumor resection. OTC was performed before postoperative chemotherapy. After 7 years, she decided to undergo OTT following the diagnosis of chemotherapy‐induced premature ovarian insufficiency. On postoperative day 104, ovarian stimulation was started, yielding one embryo after 3 days. Embryo transfer was performed during a hormone replacement cycle. At 6 weeks and 1 day, the human chorionic gonadotropin level was 81.5 mIU/mL; however, no gestational sac was observed on ultrasonography, indicating a biochemical pregnancy. Our data will be useful for the further development of fertility preservation options in Japan in the future.

## Introduction

With the rapid development in cancer treatment, fertility preservation is important when making post‐cancer treatment life plans and has attracted considerable social attention in Japan. Cryopreservation of the embryo, oocyte, and ovarian tissue is a fertility preservation method used for cancer patients whose ovarian function has been significantly reduced by the treatment of the primary disease, especially chemotherapy and radiotherapy. Embryo and oocyte cryopreservation require approximately 2 weeks from ovarian stimulation to egg retrieval; thus, ovarian tissue cryopreservation (OTC) and ovarian tissue transplantation (OTT) are the fertility preservation methods of choice for prepubertal girls and women in whom cancer treatment cannot be delayed. There are very few reports on OTT; in contrast, OTCs have been performed in more than 4500 patients worldwide and in more than 200 patients in Japan.^1^ Approximately 130 births after OTT have been reported worldwide,^2^ but none in Japan.

In our hospital, we performed 23 OTC and two OTTs, following Ethics Committee approval in January 2007. This is the first report in Japan wherein OTT was performed after premature ovarian insufficiency (POI) and implantation was achieved through in vitro fertilization (IVF).

## Case Presentation

An 18‐year‐old nulliparous woman diagnosed with Ewing sarcoma of the seventh thoracic vertebra, underwent tumor resection. The patient was informed about oocyte cryopreservation and OTC and advised of the better odds of pregnancy with oocyte cryopreservation than with OTC, and that the former did not require surgery. Since postoperative chemotherapy could not be delayed, the patient decided to undergo OTC to preserve fertility. Her menstrual cycle was regular, and she had no comorbidities. Blood hormone levels were within the normal range. Transvaginal ultrasound (TVUS) revealed no abnormalities, and she underwent laparoscopic right oophorectomy; ovarian cortical tissue was cut into 29 pieces, each measuring 5 × 5 × 1 mm, and preserved by slow freezing. She then received chemotherapy with vincristine, doxorubicin, and cyclophosphamide, alternated with ifosfamide and etoposide, for 10 months. The total dose of cyclophosphamide administered was 10.8 g/m^2^ (1.2 g/m^2^ per dose). Subsequently, the primary disease was controlled. Two months after chemotherapy, because of ovarian insufficiency, her follicle‐stimulating hormone (FSH) and estradiol (E2) levels were 127.1 mIU/mL and <10 pg/mL, respectively. Therefore, hormone replacement therapy (HRT) was initiated with 0.72 mg of E2 (transdermal, on alternate days) and 10 mg of oral dydrogesterone (for 12 days).

**FIGURE 1:**
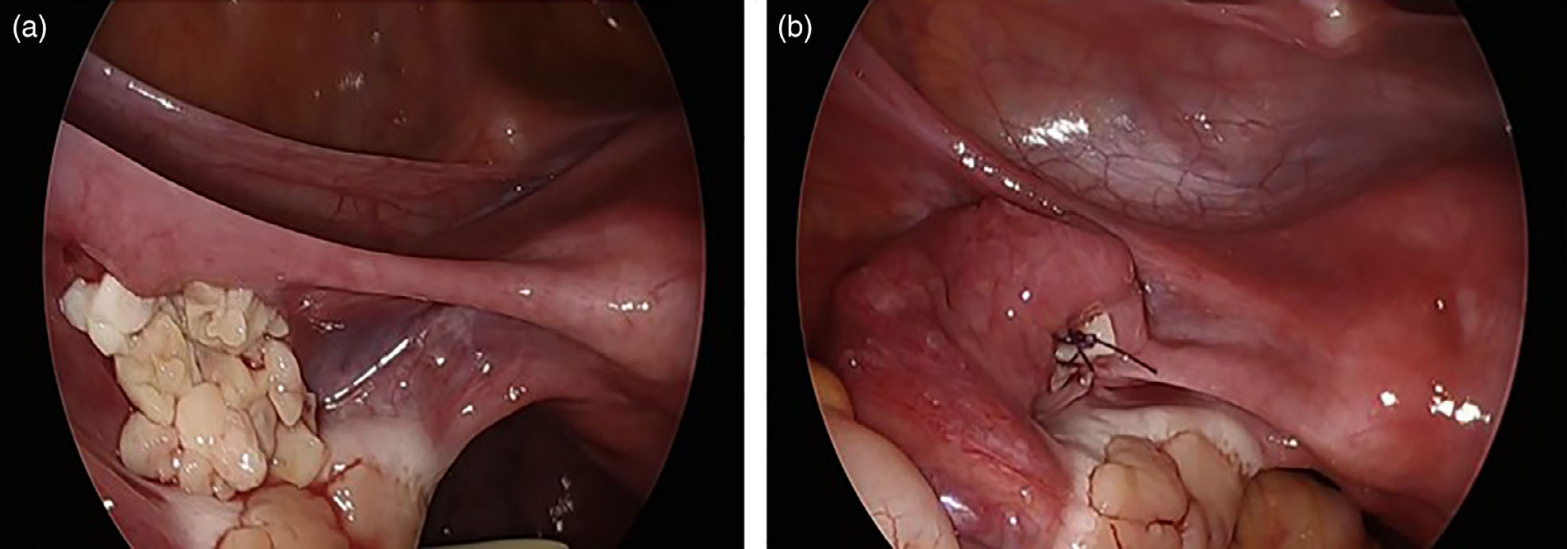
First transplant. Ten pieces of ovarian tissue are placed in the left mesosalpinx (a), and the peritoneum is sutured (b)

Seven years after her first visit, at age 25 years, she got married, and HRT was discontinued. One month later, she experienced amenorrhea, and her hormone levels at that time were as follows: FSH, 131.1 mIU/mL; E2, <10 pg/mL; and anti‐Mullerian hormone (AMH), 0.03 ng/mL. These results indicated POI; therefore, she decided to undergo OTT. The transplantation site was in the left mesosalpinx, and 10 pieces were transplanted using 5–0 absorbable sutures (Figure 1). The preoperative HRT regimen was resumed postoperatively. Furthermore, we monitored her ovarian function through blood examinations and abdominal ultrasounds weekly or fortnightly. If a follicle appeared or estrogen levels increased, we monitored her more closely. By postoperative day 64, a 14‐mm follicle was found on TVUS in the implanted left mesosalpinx without ovarian stimulation, and the patient's serum E2 level increased to 117 pg/mL. Consequently, HRT was continued, but the follicle did not develop, and pregnancy did not occur. At 13 months postoperatively, ovarian stimulation was performed; however, no follicular development was observed, leading to treatment discontinuation.

**FIGURE 2:**
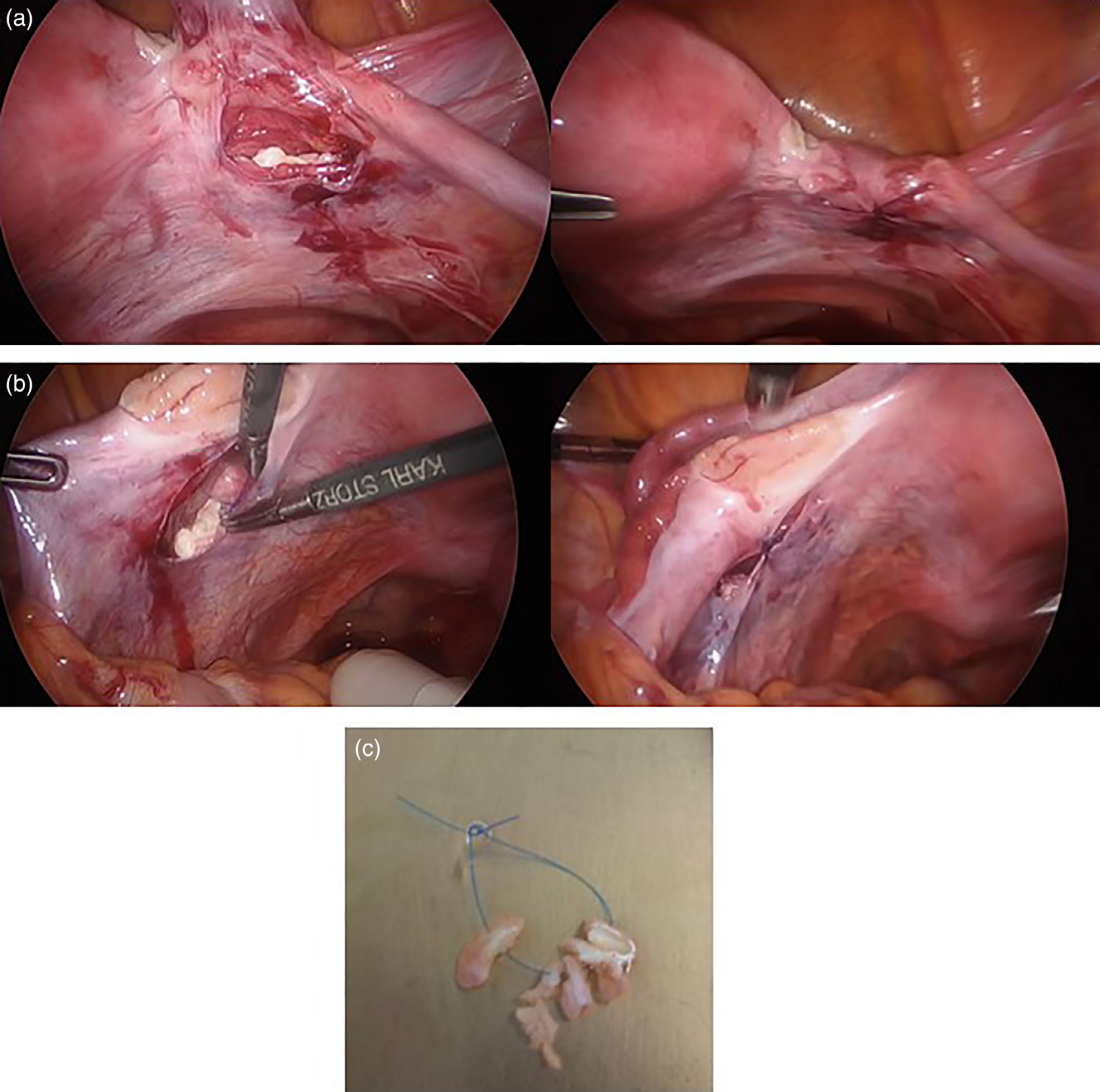
Second transplant. On both sides, 2‐cm incisions are made in the parietal peritoneum below the ovary. A subperitoneal pocket is dissected, and each of the four tissue pieces is placed (a, b). Ovarian tissues are connected in a bead‐like pattern using 5–0 absorbable sutures (c)

**FIGURE 3:**
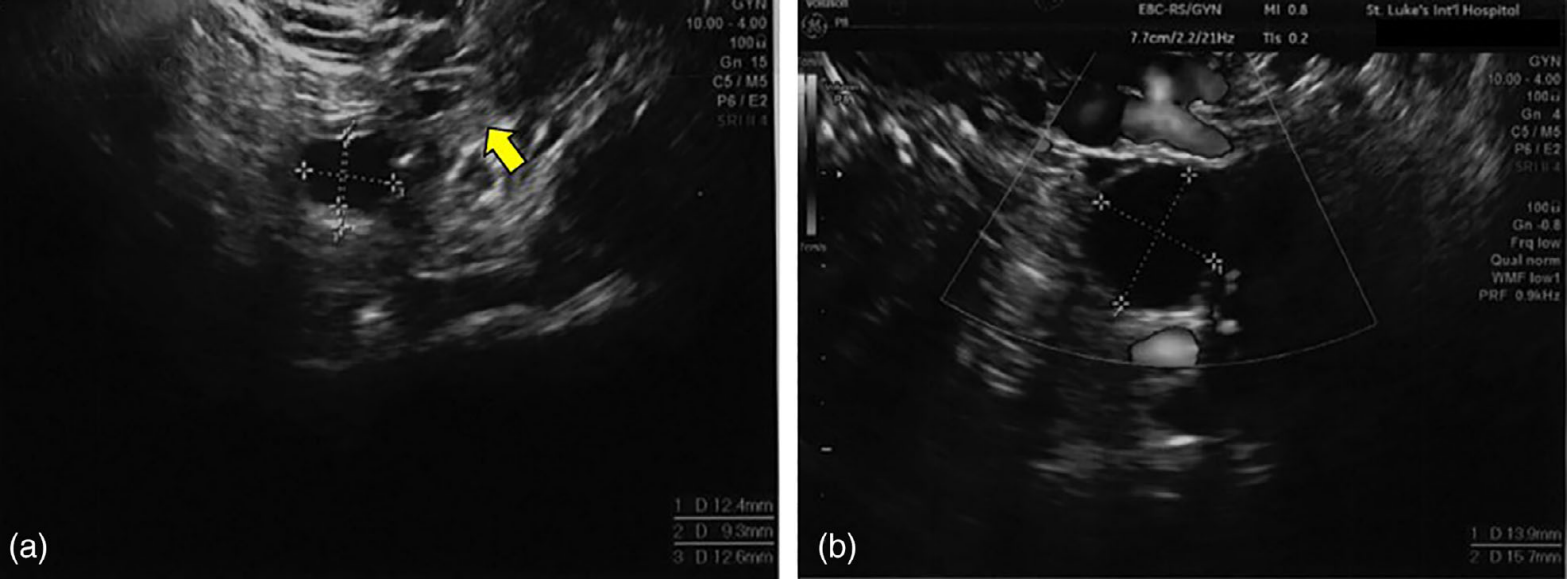
Follicular development findings by ultrasound. A follicle is seen outside the remaining left ovary (yellow arrow) (a); it continues to develop till the next day (b)

A second OTT was performed 15 months after the first due to poor response to ovarian stimulation. In the abdominal cavity, there were slight filmy adhesions around the region of the right ovary, post‐oophorectomy, but no other findings were noted. Bilaterally, a 2‐cm incision was made in the parietal peritoneum below the ovary. A subperitoneal pocket was dissected, and the four pieces of tissue were connected in a bead‐like pattern, using 5–0 absorbable sutures (Figure 2). At the time of transplantation, we did not consider the position of the blood vessels. After the second transplant, HRT was recommenced. On the 104th day postoperatively, ovarian stimulation with 150 IU of human menopausal gonadotropin (hMG) was performed for 9 days; due to poor response, the dose was increased to 300 IU on day 10. On day 21 of stimulation, an 11‐mm ovarian follicle was detected by TVUS in the left peritoneal pocket, and her serum E2 level was 180 pg/mL (Figure 3). When the diameter of the follicle increased to 14 mm, administration of gonadotropin‐releasing hormone (GnRH) antagonist (0.25 mg/day) was started, and hMG administration (300 IU/day) was continued. On day 25, when the follicle's diameter reached 17 mm, 5000 IU of human chorionic gonadotropin (hCG) was administered intramuscularly, and transvaginal oocyte retrieval was performed 35 h thereafter. With TVUS, we could access the follicle by pressing the probe against the vaginal wall; this reduces the distance between the follicle and the probe. However, caution was taken to avoid puncturing the intestine and blood vessels. One oocyte was fertilized in vitro, yielding one embryo on day 3 (Veeck classification: eight cells grade 2). Because of menstrual irregularities, a combined oral contraceptive (0.05 mg of ethinylestradiol and 0.5 mg of norgestrel) was administered for 14 days, and from day 2 following withdrawal bleeding, endometrial adjustment was performed in an HR cycle. Endometrial preparation was achieved on day 3 of the HR cycle by a step‐up regimen using transdermal estradiol (1.44–4.32 mg/day). Vaginal micronized progesterone (300 mg/day) was added as luteal support on day 16 when the E2 level was 422 pg/mL. On day 19, embryo transfer was performed, and the above‐listed medications were continued. Pregnancy was confirmed on day 39. On day 46 (6 weeks and 1 day of gestation), no gestational sac was observed, and the hCG level was 81.5 mIU/mL. Subsequently, the hCG level decreased, confirming a biochemical pregnancy.

This study conforms with the Declaration of Helsinki and was approved by the Ethics Committee of our institution. Written informed consent was obtained from the patient included in the study.

## Discussion

OTT has resulted in 130 births worldwide,^2^ and this number is expected to exceed 200.^3^ However, there have been no reports of births or pregnancies following OTT in Japan. This is due to the extremely small number of cases of OTC, with only 3%–5% of frozen ovaries actually transplanted.^4^ In 2005, the world's first birth following OTT was reported. In the same year, the Ethics Committee of Okayama University approved this method for the first time in Japan; our hospital received the second earliest approval in 2007. This is the first report of implantation following OTT, and we believe that this is partly due to its early clinical introduction in our patient.

Since most of the reported childbirths resulted from orthotopic transplants,^5^ we also chose this technique. In this case, the first transplant was performed in the left mesosalpinx and the second in the subperitoneal pocket below the ovary on both sides. FertiPROTEKT, a fertility preservation network extending to about 100 institutions in Germany, Austria, and Switzerland, recommends implantation into the peritoneal pocket because of the simplicity of the procedure.^6^ Our first choice is to transfer the ovarian tissue into the mesosalpinx, which has abundant blood flow and less risk of organ damage during egg retrieval. However, in some cases, we may use a peritoneal pocket because adhesions might occur after oophorectomy or multiple transplants. Herein, it is recommended that the peritoneum be deployed just below the ovary as a landmark, considering the fact that follicle growth would have to be monitored and that egg retrieval would have to be performed after transplantation.

Moreover, there have been multiple reports on the amount of tissue to be transplanted. We used 10/29 (34%) ovarian tissue fragments in the left mesosalpinx for the first transplant and 8/29 (27.6%) for the second—four on each side of the peritoneal pocket. The area in the mesosalpinx available for implantation was narrow, and the tissue fragments might have overlapped, resulting in partial blood flow impairment. Thus, the amount of tissue transplanted was reduced in the second procedure, and the tissue fragments were connected in a bead‐like pattern using 5–0 absorbable sutures. Some reports recommend the use of 15%–20% of the total ovarian tissue,^6^ but in cases of poor ovarian function before OTC, the number of follicles in the tissue is expected to be low. The patient's AMH level before transplantation was 0.02 ng/mL, and the ovarian reserve was very low; therefore, we decided to transplant a larger number of grafts based on our prior discussion (on the possible outcomes of OTT) with the patient. It is important to assess the ovarian reserve before OTC and accordingly consider tissue volume and retransplantation. Since ovarian reserve after OTT is much worse than common infertility patients, optimal ovarian stimulation after OTT remains to be elucidated.

We started ovarian stimulation by 150 IU/day hMG because we detected only one antral follicle. However, we should probably have started ovarian stimulation by 300 IU/day in order to maximize the ovarian response.

It has been reported that transplanted ovarian tissue recovers its function in 2–5 months, and that in cases of poor response after 6 months of observation postoperatively, retransplantation should be considered.^7^ Therefore, we performed retransplantation at 15 months after the first transplantation. While it is reported that the average lifespan is 4 or 5 years,^8^ it should also be recognized that it can range from <1 year to >10 years in individual cases.^5^ In the present case, since the patient continued to have low estrogen levels 2 weeks after OTT, HRT was initiated due to concerns about bone density; however, this was unnecessary considering that it generally takes several months for ovarian tissue to recover its function.

When choosing OTT, clinicians should be aware of minimal residual disease and explain the possibility of recurrence to patients. At our institution, we also excluded malignancy by histopathological evaluation of the ovarian cortex and part of the medulla at the time of OTC. Although in a previous study, none of the cases of recurrence after OTT was caused by reimplantation,^9^ we need to carefully follow up the patients in case of recurrence.

This is the first report in Japan of a biochemical pregnancy resulting from OTT performed for POI. Considering the number of births worldwide, further investigation of this technique is required. In particular, transplant location, tissue volume, freezing and thawing methods, and regulated ovarian stimulation must be evaluated. The Japanese government plans to subsidize fertility preservation options, including OTT, in 2021; thus, understanding these cases is important.

Herein, we have described egg retrieval, embryo transfer, and implantation after OTT. Our data are useful for the future development of fertility preservation options in Japan.

### Conflict of Interest

None declared.

## Author Contributions

All authors critically revised the report, commented on drafts of the manuscript, and approved the final report.

## Data Availability

The data that support the findings of this study are available on request from the corresponding author. The data are not publicly available due to privacy or ethical restrictions.
